# 661. Mortality Rates and Resistance Profiles of Salmonella spp. Infections: Implications for Clinical Practice and Public Health Interventions

**DOI:** 10.1093/ofid/ofad500.724

**Published:** 2023-11-27

**Authors:** Fernando Rosso, David E Rebellon-Sanchez, Julio Llanos-Torres, Carolina Álvarez-Ortega, Leidy J Hurtado-Bermudez

**Affiliations:** Fundación Valle del Lili, Cali, Valle del Cauca, Colombia; Fundacion Valle del Lili, Cali, Valle del Cauca, Colombia; Fundacion Valle del Lili, Cali, Valle del Cauca, Colombia; Fundación Valle del Lili, Cali, Valle del Cauca, Colombia; Fundación Valle del Lili, Cali, Valle del Cauca, Colombia

## Abstract

**Background:**

Salmonellosis is a frequent foodborne infection. There is not much information about the mortality and resistance profiles of Salmonella spp. infections in Latin America. The aim of the study was to determine overall mortality, related risk factors, and antibiotic resistance in patients with Salmonella infection attended at university hospital of Colombia between 2012 - 2021

**Methods:**

Retrospective observational study. All patients with a microbiological confirmed diagnosis of Salmonella spp. were included. The sociodemographic, clinical and microbiological characteristics were described. Log binomial regression models were used to establish factors associated with mortality

**Results:**

A total of 522 patients were included, 54.8% were females, the median age was 16 years (IQR= 1-50). Mortality occurs in 4.02% of the patients. The proportion of deaths was higher in patients with bacteremia (RR = 3.65, 95% CI 1.59 - 8.40) hematologic malignancy (RR = 2.39, 95% CI 0.90 - 6.29) and those who require treatment in the intensive care unit (ICU) (RR=7.92, 95% CI 3.14 -19.97). There was no significant difference in mortality among those younger than 15 years old than older (2.8% vs. 5.4%, p = 0.13). Factors associated with mortality were bacteremia (aPR = 3.41 CI95%, 1.08 - 10.76) and requirement of ICU (aPR = 8.13 CI95%, 1.82 - 37.76). An increase in resistance rates over the study period (trend chi2: p< 0.05). Increment in resistance was observed against ciprofloxacin (from less than 1% to 60%) and ampicillin/sulbactam (from 10.9% to 50%) (Fig. 1). No association was found between salmonella susceptibility and mortality

**Figure:**
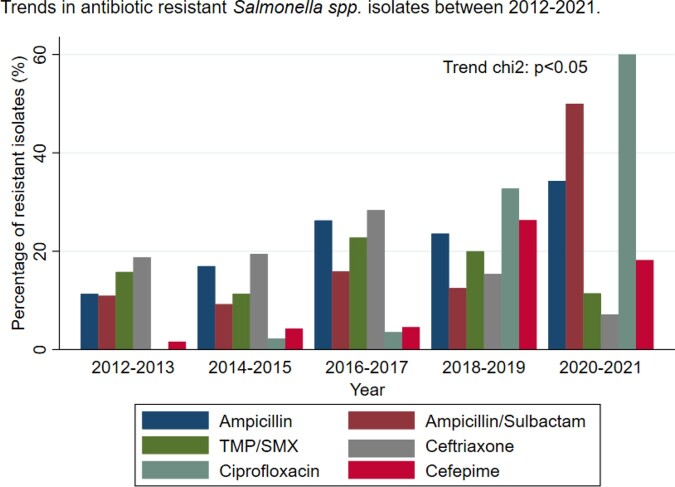

**Conclusion:**

Despite being a preventable and treatable disease, Salmonellosis still poses a public health concern. Our study highlights the persistence of mortality due to this condition and a significant increase in antibiotic resistance rates over the last decade. Vulnerable patient groups showed higher mortality rates

**Disclosures:**

**All Authors**: No reported disclosures

